# Increased Glyoxalase-1 Levels in *Fkbp5* Knockout Mice Caused by Glyoxalase-1 Gene Duplication

**DOI:** 10.1534/g3.113.006445

**Published:** 2013-08-01

**Authors:** Lorenz K. Kollmannsberger, Nils C. Gassen, Andrea Bultmann, Jakob Hartmann, Peter Weber, Mathias V. Schmidt, Theo Rein

**Affiliations:** Max Planck Institute of Psychiatry, 80804 Munich, Germany

**Keywords:** FKBP51, flanking gene problem, glyoxalase-1, knockout mice

## Abstract

*Fkbp5* is genetically linked to stress-related diseases. *Fkbp5* knockout mice are available and widely used to explore the role of *Fkbp5* in health and disease. We found that these mice carry a gene duplication of glyoxylase-1, which explains why glyoxylase-1 levels are increased in the *Fkbp5* knockout mice.

In several genetic studies researchers linked FK506 binding protein 5 (*Fkbp5*) to stress-related diseases and phenotypes such as major depression, posttraumatic stress disorder, and recovery from psychosocial stress (Binder *et al.* 2004; Zimmermann *et al.* 2011; Klengel *et al.* 2013). In addition, *Fkbp5* is also linked to treatment response in depression (Binder *et al.* 2004; Lekman *et al.* 2008). To elucidate the role of FKBP5 in an animal model, a conventional knockout mouse has been constructed and made available to the scientific community (Tranguch *et al.* 2005; Touma *et al.* 2011). These *Fkbp5*-deficient mice show no overt phenotype unless they are older than 10 months of age (O’Leary *et al.* 2011) or exposed to stress (Touma *et al.* 2011; Hartmann *et al.* 2012).

To elucidate the effects of *Fkbp5*-deletion on molecular pathways, we compared the expression profile of *Fkbp5^+/+^* and *Fkbp5^−/−^* litter mates. A marked difference in glyoxalase-1 (*Glo1*) mRNA was observed with *Fkbp5^−/−^* mice expressing greater levels (not shown). Consistent with this observation, about 2-fold more GLO1 protein was found in *Fkbp5^−/−^* mice (Figure 1A). For more detailed molecular analyses, we sought to establish a cellular model. Therefore, we overexpressed FKBP5 by transient transfection in either primary rat astrocytes or HEK293 cells. However, overexpression of FKBP5 did not change *Glo1* mRNA (not shown) and also not alter protein levels of GLO1 (Figure 1B).

**Figure 1:**
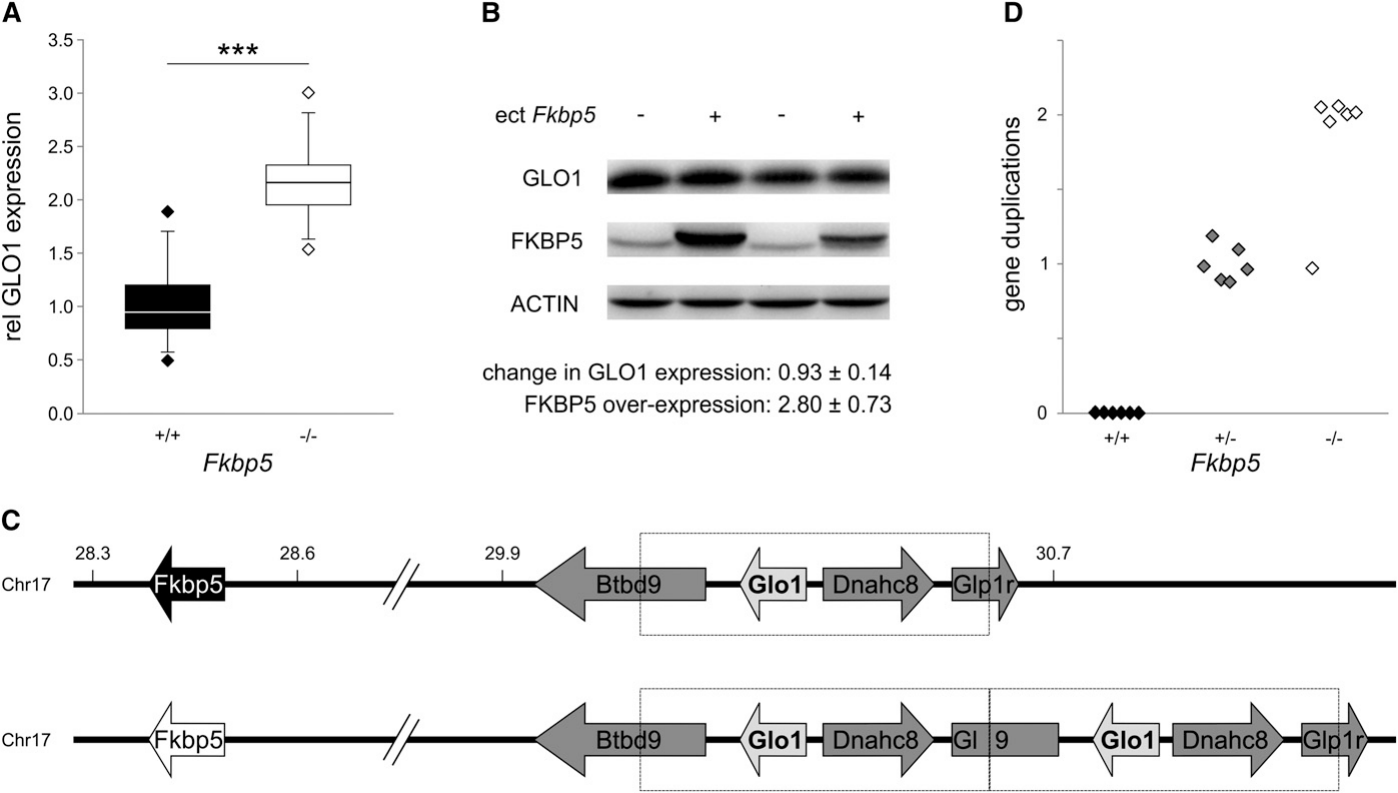
(A) Comparison of GLO1 protein expression in hippocampi from *Fkbp5^−/−^* and *Fkbp5^+/+^* mice. Hippocampi were prepared, and GLO1 expression was determined after protein extraction by Western blotting (polyclonal antibody; Santa Cruz Biotechnologies); signals were normalized to ACTIN (polyclonal antibody; Santa Cruz Biotechnologies). Expression difference was analyzed by Tukey’s test (n = 12 per genotype; *P* < 0.001). (B) Overexpression of *Fkbp5* in HEK-293 cells by transient transfection did not affect GLO1 levels. Cells were transfected with *Fkbp5* expressing or control vector, and protein levels were determined in cell extracts by Western blotting 3 d later. Mean protein levels ± SEM of GLO1 and FKBP5 (polyclonal antibody; Bethyl Laboratories) normalized to ACTIN are indicated (n = 5). (C) Scheme of genomic arrangement of *Fkbp5* (28.5−28.4 Mb) and *Glo1* (30.6−30.6 Mb) on chromosome 17 (28.3−30.7 Mb), without (upper) and with (lower) *Glo1* gene duplication. The wild-type *Fkbp5* allele (originating from C57BL/6 mice) is usually coinherited with a single copy of *Glo1*, whereas the knockout *Fkbp5* allele (originating from 129SvJ mice) is coinherited with two copies of *Glo1*. (D) Verification of coinheritance of the *Fkbp5* knockout allele with *Glo1* duplication. Genomic duplications of the *Glo1* spanning region were determined by quantitative reverse-transcription PCR (two independent PCRs per mouse) with primers against the duplication transition region [fw 5′-CTCTGCCCCAGAGAACAGTC and rv 5′-TGATAGAGGCCACACAGCAG (Williams *et al.* 2009)] and normalized to genomic levels of *Npsr1* (determined by quantitative reverse-transcription PCR with the following primers: fw 5′-CAGCTGCTGCCCCGGCTAAC and rv 5′-GGTTGGCTGGCATGGCTCAGG).

We noted that the genes *Fkbp5* and *Glo1* are only approximately 2 Mb apart from each other on chromosome 17 of the mouse (Figure 1C). In addition, gene duplication around *Glo1* was observed in several mouse strains (Egan *et al.* 2007; Williams *et al.* 2009). The *Fkbp5* deletion was constructed in 129SvJ ES cells, and the resulting mice were then crossed with C57BL/6 animals; 129SvJ mice carry the *Glo1* gene duplication but C57BL/6 mice do not (Williams *et al.* 2009).

Therefore, it appeared likely that through selection of *Fkbp5^+/+^* and *Fkbp5^−/−^* alleles in the subsequent crossings the *Glo1* gene duplication originating from 129SvJ mice was coselected with the *Fkbp5^−/−^* allele, whereas the unduplicated *Glo1* cosegregated with the *Fkbp5^+/+^* allele. To test this hypothesis, we used polymerase chain reaction (PCR) primers designed for monitoring the *Glo1* gene duplication (Williams *et al.* 2009). DNA samples from *Fkbp5^−/−^*, *Fkbp5^-/+^* and *Fkbp5^+/+^* mice were probed. No *Glo1* gene duplication was detectable in *Fkbp5^+/+^* mice, whereas the PCR signal in *Fkbp5^−/−^* mice was clearly detectable and twice as high as in *Fkbp5^-/+^* mice (Figure 1D). Therefore, the greater levels of mRNA and protein of GLO1 in *Fkbp5^−/−^* mice compared with wild-type mice are likely due to the double *Glo1* gene dose in these mice. In general, this so-called “flanking allele” problem is a well-known and likely common phenomenon in gene knockout via homologous recombination (Gerlai 1996; Crusio *et al.* 2009). It could be avoided, for example, by genome editing with engineered nucleases or by using inducible gene knock out techniques (Sauer 1998; Carbery *et al.* 2010).

GLO1 is a ubiquitously expressed enzyme involved in the detoxification of methylglyoxal (Thornalley 2008). Methylglyoxal is a toxic byproduct of glycolysis that leads to protein modification and apoptosis (Thornalley 2008) and influences behavior when acting as GABA_A_ receptor agonist (Distler *et al.* 2012). GLO1 has been linked to diabetic complications, anxiety disorders, schizophrenia, seizure susceptibility, pain, cancer, and aging (Thornalley 2008; Distler and Palmer 2012). At least some of these diseases and phenotypes also have been associated with *Fkbp5*, making *Fkbp5^−/−^* mice potentially very useful genetic model for further investigation. Our observation of *Glo1* gene duplication in *Fkbp5^−/−^* mice suggests that the *Glo1* status should be taken into consideration when interpreting data. Studies on neuroendocrine and stress effects of *Fkbp5* gene deletion published so far are likely not biased by the *Glo1* gene duplication, in particular because no differences between *Fkbp5^+/+^* and *Fkbp5^−/−^* mice have been observed under basal conditions when neuroendocrine parameters or behavior, including anxiety-like behavior, is assessed (Touma *et al.* 2011; Hartmann *et al.* 2012).
